# 2D/3D Microanalysis by Energy Dispersive X-ray Absorption Spectroscopy Tomography

**DOI:** 10.1038/s41598-017-16345-x

**Published:** 2017-11-28

**Authors:** Dario Ferreira Sanchez, Alexandre S. Simionovici, Laurence Lemelle, Vera Cuartero, Olivier Mathon, Sakura Pascarelli, Anne Bonnin, Russell Shapiro, Kurt Konhauser, Daniel Grolimund, Pierre Bleuet

**Affiliations:** 1 Paul Scherrer Institut, CH-5232 Villigen PSI, Switzerland; 2 ISTerre, UGA, CNRS, Observatoire des Sciences de l’Univers, CS 40700, 38058 Grenoble, France; 3 LGL-TPE, Univ Lyon, Ens de Lyon, Univ Claude Bernard, CNRS UMR5276, F-69342, Lyon, France; 4 ESRF-The European Synchrotron, 71, Avenue des Martyrs, Grenoble, France; 5 Geological and Environmental Sciences Department, CSU Chico, Chico, CA USA; 6 Department of Earth and Atmospheric Sciences, University of Alberta, Edmonton, AB Canada; 7 University Grenoble Alpes, F-38000 Grenoble, France; 8 CEA, LETI, MINATEC Campus, F-38054 Grenoble, France

## Abstract

X-ray spectroscopic techniques have proven to be particularly useful in elucidating the molecular and electronic structural information of chemically heterogeneous and complex micro- and nano-structured materials. However, spatially resolved chemical characterization at the micrometre scale remains a challenge. Here, we report the novel hyperspectral technique of micro Energy Dispersive X-ray Absorption Spectroscopy (μED-XAS) tomography which can resolve in both 2D and 3D the spatial distribution of chemical species through the reconstruction of XANES spectra. To document the capability of the technique in resolving chemical species, we first analyse a sample containing 2–30 μm grains of various ferrous- and ferric-iron containing minerals, including hypersthene, magnetite and hematite, distributed in a light matrix of a resin. We accurately obtain the XANES spectra at the Fe K-edge of these four standards, with spatial resolution of 3 μm. Subsequently, a sample of ~1.9 billion-year-old microfossil from the Gunflint Formation in Canada is investigated, and for the first time ever, we are able to locally identify the oxidation state of iron compounds encrusting the 5 to 10 μm microfossils. Our results highlight the potential for attaining new insights into Precambrian ecosystems and the composition of Earth’s earliest life forms.

## Introduction

Synchrotron-based techniques, such as X-ray fluorescence (XRF), diffraction (XRD), absorption (XAS) and emission (XES) spectroscopies with hard X-rays have recently been used to provide non-destructive, high resolution multimodal imaging of chemical speciation for various complex, heterogeneous systems^1–9^. In this regard, the development of structural and chemical imaging in two and three dimensions has been extensively reported because it has (i) the potential for *in situ* analysis^10–13^; (ii) high resolution of the order of tens of nanometres^14^; (iii) the capability to probe tens of micrometres within the material being studied^15^; (iv) the capability to achieve depth resolved crystalline contrast through XRD analysis^2,16–18^; and (v) chemical-elemental contrast through XRF^19–22^. In the last few years, numerous X-ray imaging techniques have been developed and applied, both full-field and scanning, from the µm to the nm scale. Among the main advantages of the full-field techniques are their capability to perform high-speed 3D measurements^23^. By contrast, X-ray scanning techniques^2,6,17^ are of relatively reduced data analysis complexity and are used when hyperspectral analysis, e.g., simultaneous XRD and XRF tomography, is required^2,4–7,24,25^. This combination is of particular interest for a great variety of systems where the spatially resolved correlation between elemental composition and the crystalline structure is required to understand chemical and physical properties. Moreover, the determination of short range atomic arrangement and element-selective electronic structure by XAS analysis is crucial for the characterization and identification of chemical species.

In the case of micro XAS characterization, there remain significant challenges in the study of heterogeneous materials at the micrometre scale. Some of these are due to absorption effects, and spatial and temporal pointing instabilities of the X-ray beam. In particular, the latter arise from vibrations of beamline components and optical elements, and source instabilities. Compared to 2D and 3D μXRD and μXRF investigations, where full XRD patterns and full XRF spectra can easily be obtained in single event capture mode, XAS spectra acquisition normally demands energy scans^22,26^. By adding the energy dimension to the spatial ones, the scanning time greatly increases at the cost of spatial uncertainties attributed to temporal instabilities. An exciting alternative that overcomes these issues is the energy-dispersive XAS (ED-XAS) technique^27^. In ED-XAS, a curved crystal (also called “polychromator”) disperses and focuses a polychromatic X-ray beam onto the sample. The transmitted beam is then detected by a position sensitive detector, where the energy-direction correlation is transformed into an energy-position correlation. The main advantages of ED-XAS spectrometers with respect to energy scanning spectrometers are: (1) the speed of acquisition, since all energy points are acquired in parallel on the position sensitive detector, and (2) the stability of the energy scale and focal spot position since there are no moving components during acquisition. These allow for the collection of full XAS spectra in single shot mode with micrometre spatial resolution^28^. However, the investigation of heterogeneous materials still remains a challenge because a polychromatic fan of radiation impinges on the sample, with photons of different energies probing slightly different parts of the sample while they travel through the sample, around the focal plane. The latter is defined with a precision of ~100 microns, corresponding to typical values of depth of focus on ED-XAS beamlines. This usually limits the potential of this technique for performing spatially resolved chemical/structural analysis at the micrometre scale.

In this work, we report the application of μED-XAS tomography for 2D/3D depth-resolution of the spatial distribution of chemical species through reconstruction of the local X-ray Absorption Near Edge Spectroscopy (XANES) spectra. Experiments are carried out at the energy dispersive X-ray absorption beamline ID24 of the European Synchrotron Radiation Facility (ESRF) in Grenoble, France, a facility dedicated to fast, time-resolved and extreme-conditions X-rays absorption spectroscopy (XAS) studies^29,30^. The spatial resolution and capability for chemical speciation of this technique are investigated using a model sample containing several known iron mineral phases distributed in a light matrix, inside a pure thin-walled silica capillary, as a standard.

The technique is then applied to probe the 1.88 Ga Gunflint Iron Formation samples from the Schreiber locality in Ontario, Canada, a site made famous due to its exceptionally well preserved microfossils^31–34^. The microfossils are preserved as both carbonaceous and ferric oxide filaments and spheres, within a crystalline quartz (chert) matrix. Whether the ferric oxides are by-products of a particular metabolism of Fe(II)-oxidizing bacteria^32^ or the result of ferric iron replacement of preserved cellular structures post depositional alteration^35^ remains the subject of on-going debate. However, resolving this issue is crucial because understanding the timing of Fe(II) oxidation bares directly on the issue of the oxygenation of Earth’s early ocean and atmosphere^36^. In this regard, the Fe distribution within the carbonaceous walls of single microfossils observed in thin sections^33^ was shown as a potential means to make taxonomic determinations. State of the art laboratory absorption X-ray tomography was shown to be a non-destructive tool providing an overview of the distribution of highly absorbing isolated patches that were interpreted as pyritized clumps of microfossils^37^. Absorption and XRF tomographies using synchrotron radiation hard X-rays were shown to be useful techniques to non-destructively locate potential sites of life traces within cm-sized samples, but relatively limited to identify the life traces themselves within rocks^38^. The coupling of XRF and XAS spectroscopies with 2D scanning X-ray microscopy was thus performed to study microfossils based on their sulphur contents, as sulphur is the most abundant element that can be explored in samples thicker than the microbial cells^39,40^. Recently, 3.35 Ga Archean fossils could be analysed in XRF at several tens of nm resolution at the hundred ppm trace element levels, however, confined to intermediate Z elements^41^. In this study, we test the capability of XAS tomography to display the 3D distribution of the different iron species in sub-millimetre thick samples from the Schreiber Beach site.

## Experimental

### Synchrotron Experiments

The μED-XAS tomography experiments were conducted at the ID24 beamline^30,42^. The X-ray beam was focused horizontally and vertically to a size of about 5.5 × 2.5 (H × V FWHM) µm. The intensity transmitted by the sample is detected by a fast, low-noise, high-dynamic-range X-ray position-sensitive detector with 2048 pixels^43^. Further details on the optical scheme can be found in references Pascarelli *et al*.^30,42^.

The useful energy range of the focused polychromatic beam critically depends on the type and quality of the polychromator crystal and on the focal distance. In this experiment, we used a Si(111) crystal and a focal distance of ~0.9 m. We could focus a total energy range of ~400 eV illuminating 1300 pixels out of the total 2048. In other words, we were able to bend the polychromator to a perfect elliptical shape on roughly half of its useful length, and the slope error from this area of the crystal blurred the focal spot from the ideal 2–3 microns to about 5.5 microns FWHM.

The XAS spectra given by the product of the attenuation coefficient µ(E) by the sample path length ρ, µ(E) · ρ, were calculated pixel by pixel, or, energy by energy E, by using the Beer-Lambert law’s equation

$$\mu (E)\cdot \rho =ln{({I}_{0}/I)}_{E}$$

considering I_0_ the incident beam intensity with no sample.

**Figure 1:**
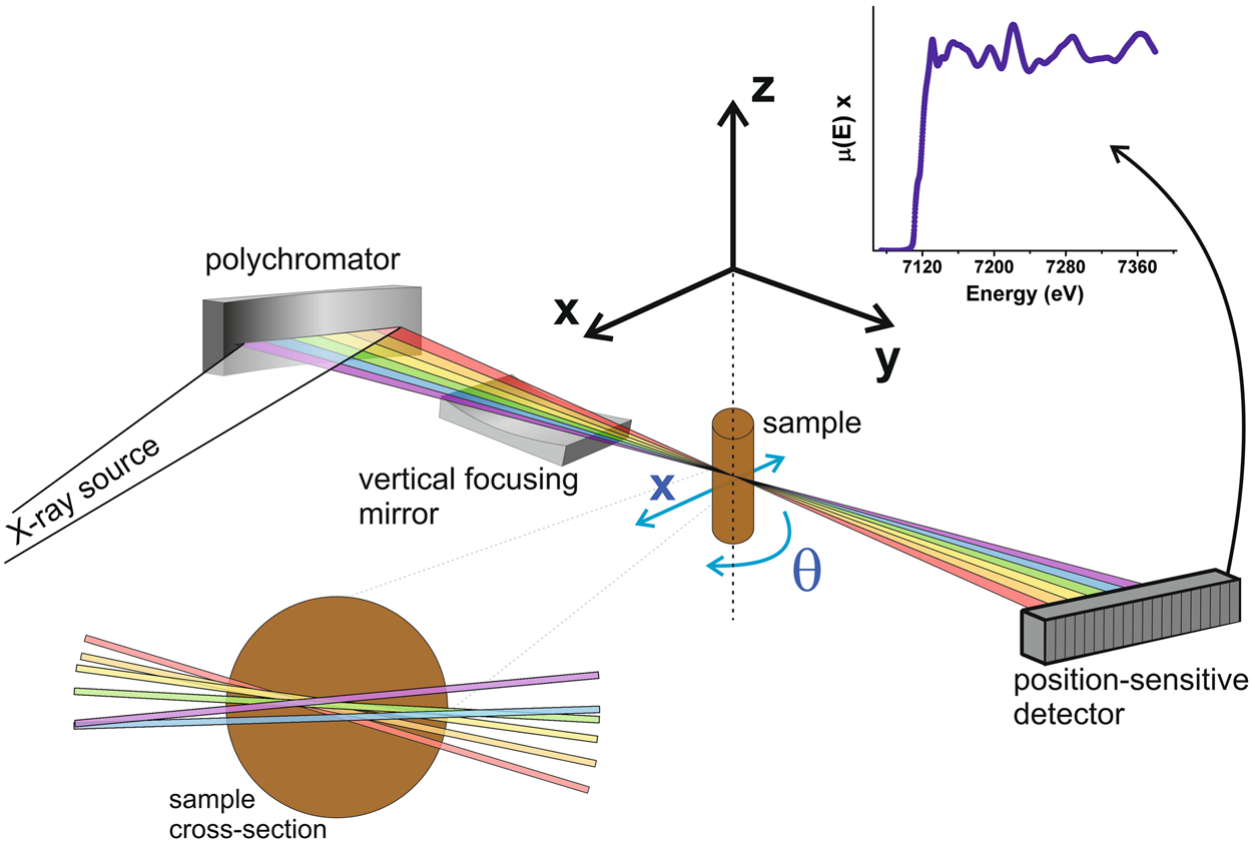
Energy-dispersive X-ray absorption spectroscopy tomography experimental setup. An incoming X-ray beam coming from an undulator source, with an energy band-width of about 1 keV is focused horizontally by a curved crystal, the polychromator, and vertically by a vertical focusing mirror onto the sample position. After the sample, the flat horizontally diverging beam is detected by a position sensitive FReLoN detector camera^43^, with which the transmitted intensities are recorded in order to calculate the spectra; in the illustration, a spectrum from metallic iron is shown. In detail on the bottom left, a cross-section of the sample illustrating the different energies of the incoming beam that transit the specimen at different angles and that are not necessarily intersected at the same region.

The sample was mounted on a motorized rotational stage and positioned in the X-ray beam focus. The tomography data acquisitions were performed by collecting XAS spectra in two dimensions: X and θ, as illustrated in Fig. 1. XAS spectra were thus collected at lateral positions across the sample, equally spaced by 1 to 2 μm, and at 90 to 180 equally-spaced angular orientations over an angle range of 180°. The sample was analysed slice by slice, where each slice consists in a 2D X by θ scan. Several slices are measured in order to obtain the full 3D sample imaging.

## Results and Discussion

### The model system: iron containing phases

**Figure 2:**
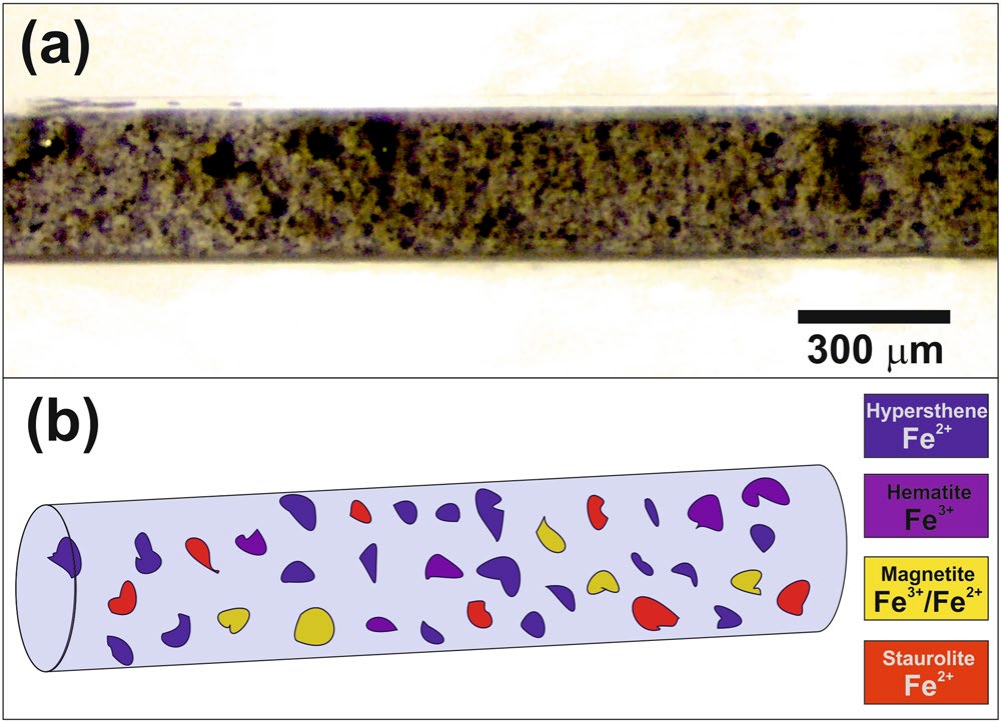
(**a**) Optical microscopy image of the glass capillary sample containing a mixture of grains of about 2–30 μm in size of four different iron containing phases: magnetite, hematite, hypersthene and staurolite, as illustrated in (**b**).

To demonstrate the capability of the technique in resolving chemical species, a sample containing several iron-bearing mineral phases was selected as a standard. The selected phases were (i) staurolite, an iron aluminium silicate containing Fe(II); (ii) magnetite, an iron oxide with Fe(II) and Fe(III), (iii) hematite, an iron oxide with only Fe(III); and (iv) hypersthene, an iron aluminium silicate containing Fe(II). The compounds, in the form of 2 to 30 μm in size grains, were mixed with an organic UV-hardening glue and placed inside a 300 µm diameter silica glass capillary with 10–20 µm wall-thickness. An optical microscopy image of the sample is shown in Fig. 2.

**Figure 3:**
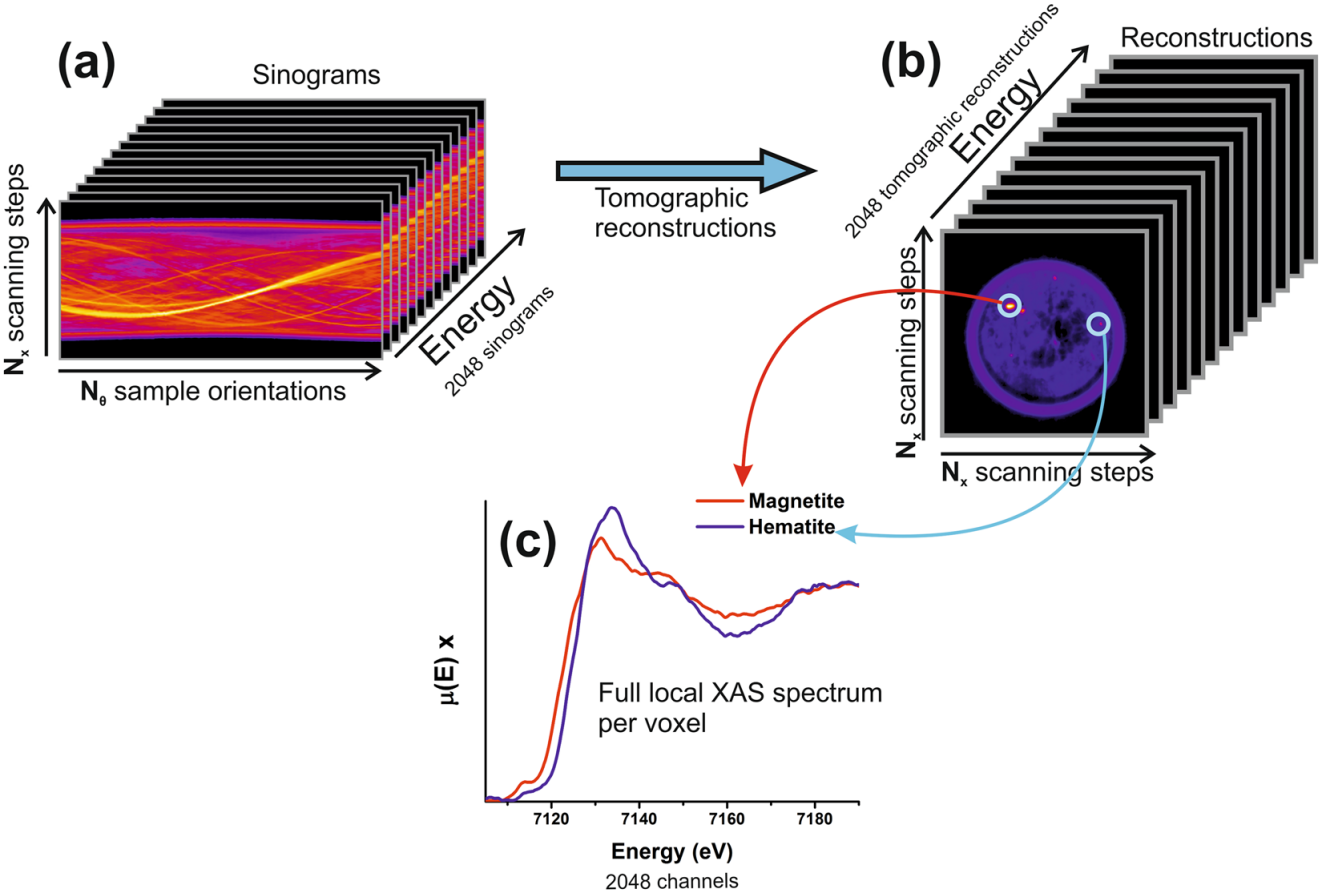
Dataset analysis workflow. Detector pixel by pixel, (**a**) 2D maps of N_X_ × N_θ_, called sinograms are build. (**b**) Through tomographic reconstructions, images of N_X_ × N_X_ of size with the µ(E)ρ distribution along the slice are obtained, for all energy steps in a stack. (**c**) For each voxel in the stack, the local XAS spectra are obtained; in this illustration, spectra from hematite and magnetite extracted from single voxels are compared.

The collected dataset consists of N_E_ 2D cartographies of N_X_ × N_θ_, called sinograms, where N_E_ is the number of detector pixels, each corresponding to an energy slice of the spectrum; N_X_ is the number of scanned lateral positions; and N_θ_ is the number of scanned sample rotations around the vertical axis. The sample was analysed slice by slice along the capillary axial length (Z direction); tomograms at different Z sample positions were collected, which encompass the third spatial dimension. The dataset was analysed similarly to μXRD and μXRF scanning tomography data sets^2,21,25,44^. Our acquired XAS spectra were collected pixel by pixel using N_E_ = 1300 out of the total 2048 pixels of the FReLoN CCD detector. For each CCD pixel, which collects an energy bandwidth of 0.2–0.3 eV, a sinogram was built. Each sinogram consists of a N_X_ × N_θ_ map, as illustrated in Fig. 3a, so that one sinogram can be built for each pixel of the CCD detector over the incident X-ray energy range. Tomographic reconstructions performed for each sinogram provide the reconstructed figures (N_X_ × N_X_ in size) as shown in Fig. 3b.

μED-XAS tomographic reconstruction has to deal with a number of issues, some of which common to XRD and XRF tomography and some which are ED-XAS specific. The photon intensity distribution in space is affected by interaction with the beamline components and optical elements (windows, mirrors and, mainly, the polychromator), which leads to significant intensity heterogeneities. Along the vertical Z direction (see coordinates in Fig. 1), these are efficiently averaged and smoothed out over the full vertical size of the beam footprint on the detector. However, this does not occur in the horizontal X direction. Different energies of the incoming beam transit the specimen at different angles, and thus, each energy probes different regions of the sample. In the ideal case of perfect optics (perfect ellipse, zero slope error) and perfect sample alignment (centre of sample on focal plane), the trajectories of X-rays of different energy cross each other at the focal plane, situated in the centre of the sample volume, and defined within the depth of focus of approximately 100 μm. However, in a real world this is never the case. In μED-XAS tomographic reconstruction there is a non-trivial issue resulting from the fact that the various trajectories do not necessarily intersect the same region of the specimen. This is due to the combination of three effects: (1) the various energies transit the specimen at different angles, (2) small deviations of sample position from the focal plane and (3) even with a perfect elliptical shape (to zero order) in the used portion of the polychromator crystal, small deviations from this shape due to slope error result in slight deviations in the trajectories of the various energies, responsible also for the blurring of the focal spot (which is larger than the intrinsic limit of 2–3 μm FWHM). A qualitative illustration is shown in Fig. 1, and in the Supplementary Material as well. The beam footprint on the position sensitive detector is sampled over the pixels of the CCD detectors, and the tomographic reconstructions obtained by each one of these pixels, which correspond to different energies, can be treated as an independent measurement. In order to correlate the same element of volume along this collection of independent tomographic reconstructions, each reconstruction is subject to a rigid-body motion (rotation and translation). This allows to match the reconstructed images with respect to the first one by using image correlation methods for a given slice (a given Z sample position), similar to those illustrated in Fig. 3b. Here, we have used two plugins of the public domain ImageJ package: TurboReg to align two images and StackReg to drive the whole stack operation (http://bigwww.epfl.ch/thevenaz/stackreg)^45^. Following this procedure, good quality XAS spectra were obtained per reconstructed voxel. Figure 3c illustrates the XANES spectra of two isolated particles of a few microns in size, where hematite and magnetite can be clearly identified.

**Figure 4:**
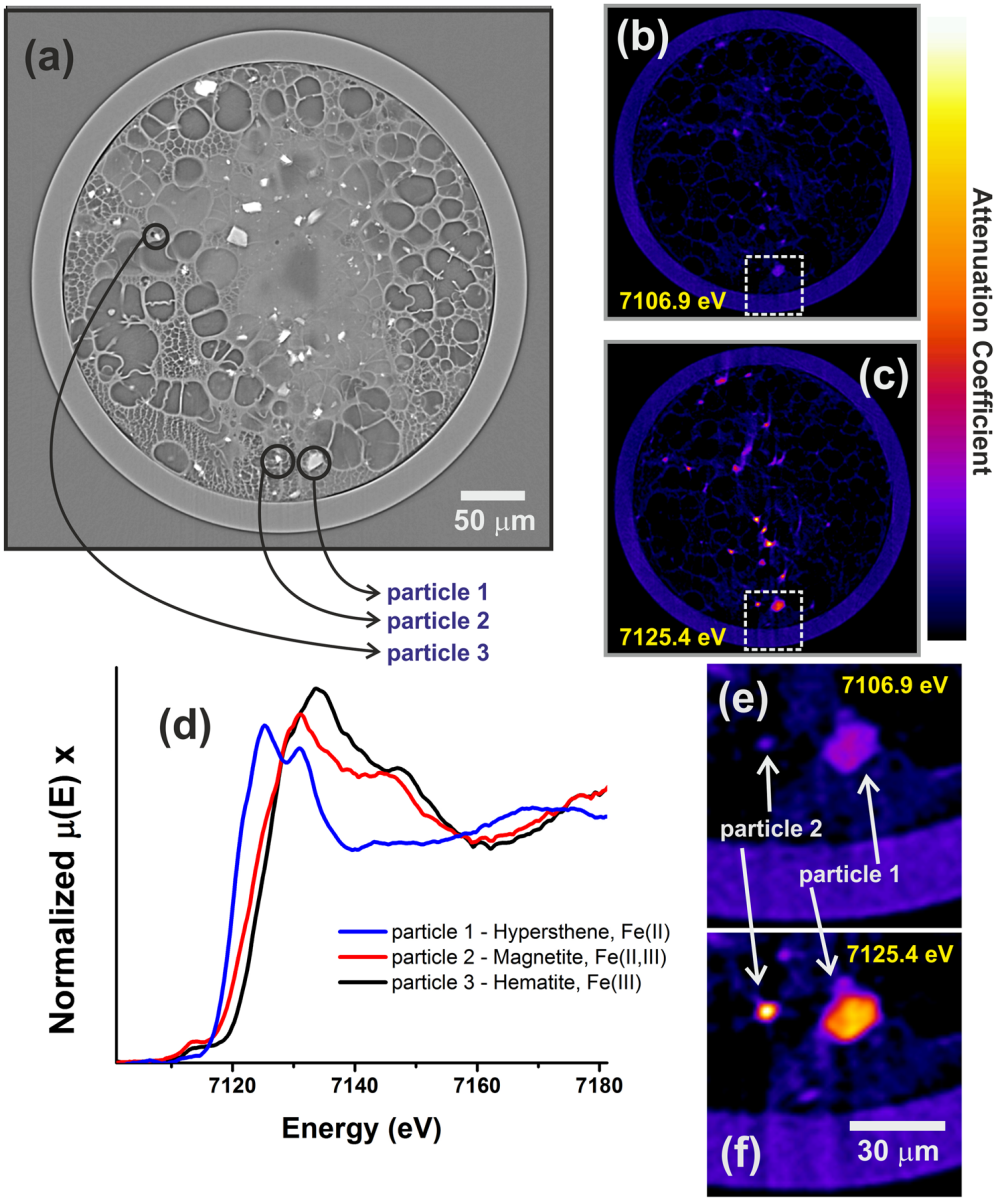
For the glass capillary sample containing several iron species, (**a**) a reconstructed slice from the full field microtomography experiments using an X-ray energy of 20 keV; the same slice reconstructed through μED-XAS tomography for two different energies: (**b**) below the iron K edge and (**c**) at 7125.4 eV, and, (**d**) single-voxel XAS spectra of the three particles indicated in (**a**), which are identified as hypersthene, magnetite and hematite. In (**e**) and (**f**), a detailed view corresponding to the dashed rectangles in figures (**b**) and (**c**).

Figure 4 compares a tomographic slice by μED-XAS (Fig. 4b,c) to those from absorption X-ray synchrotron microtomography (XCT) scans performed at the TOMCAT (Fig. 4a) beamline where 10 times higher spatial resolution is achievable. The reconstructed slices are shown in Fig. 4b,c, corresponding to energies of 7106.9 eV and 7125.4 eV, respectively. More data are provided in the Supplementary Material. Different contrast patterns are observed by comparing these two images. By considering all obtained images corresponding to different energies, the XAS spectra per voxel can be obtained. As an example, three particles are indicated in Fig. 4a, while the corresponding XAS spectra for each individual particle, obtained from single voxels, are shown in Fig. 4d. The identification of these three iron species is then readily confirmed as hypersthene, magnetite and hematite, the known mineral standards. The spatial resolution on the μED-XAS tomographic reconstructions was calculated by two different methods: (1) analysing sharp features observed on several scanned slices of different samples, which results in a resolution of about 3 μm, and (2) through Fourier Ring Correlation^46^, by analyzing several reconstructed images of different neighbouring energies in the same sample slice, resulting in a resolution of about 3.2–3.8 µm. For more details, see Supplementary Material. Figure 4e,f provide more detailed views of the corresponding dashed areas in Fig. 4b,c, and illustrate the resolving power of the technique.

The capillary sample containing different iron species can be considered as a model system for μED-XAS tomography because: (A) it is a relatively large sample (lateral size approximately 150 times larger than the observed spatial resolution); (B) it is mostly composed of a light matrix material (basically SiO_2_ thin wall capillary and the organic UV-hardening glue); and (C) it contains small (~one to few tens of μm highly localized compounds of interest containing at least a few at.% of the investigated element, in this case, iron. Condition (A) establishes the interest of imaging the system by scanning tomography; condition (B) is related to the previous condition establishing that the sample cannot be much larger than the absorption length; while condition (C) is related to condition (B) in the context of delivering sufficient absorption contrast, i.e., difference in the absorption µ(E)ρ above and below the edge (also called “jump”).

### The iron containing phases associated with Gunflint’s Microfossils

**Figure 5:**
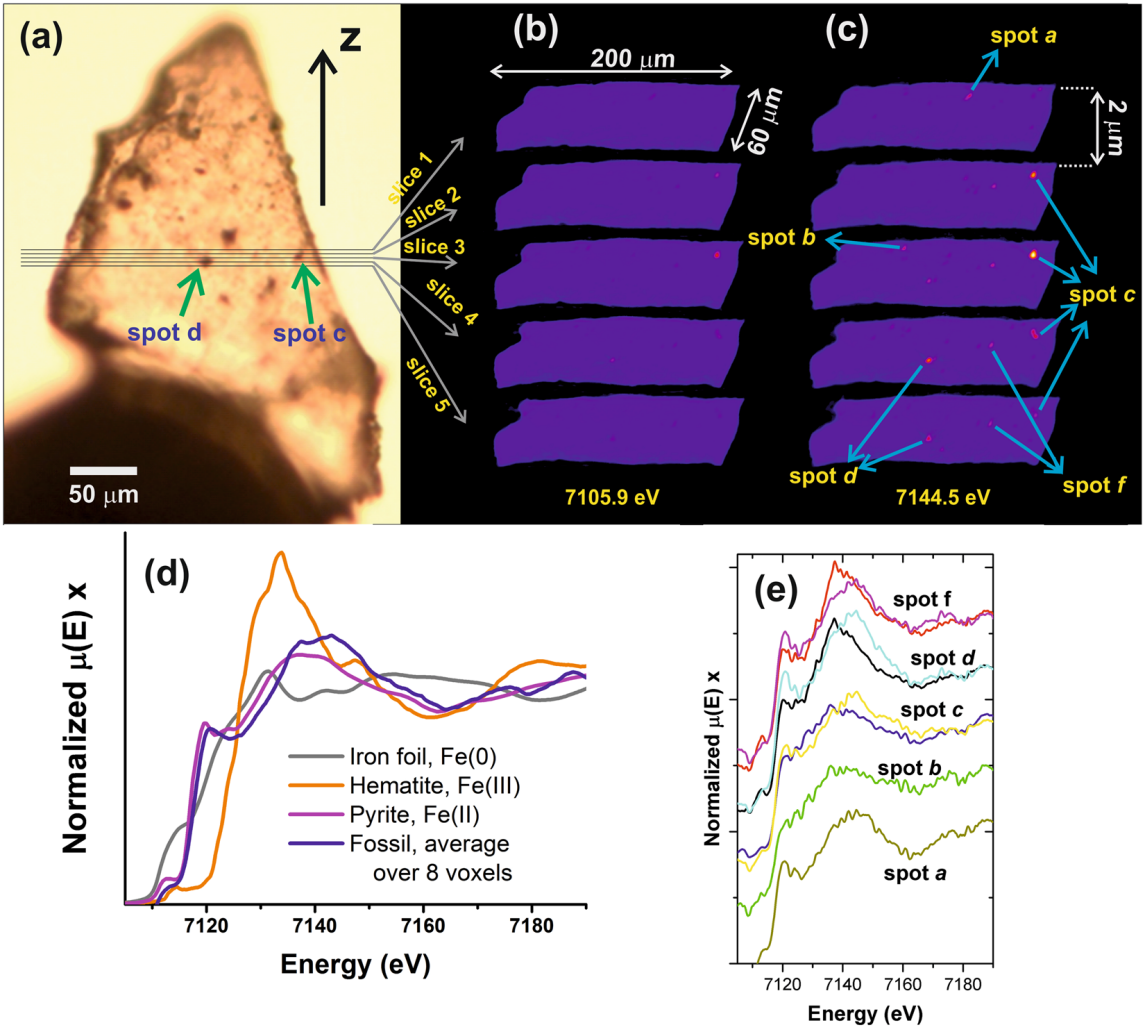
(**a**) An optical microscopy image of the investigated fossil specimen, where the five slices analysed through μED-XAS tomography experiments are indicated. Two dark inclusions are pointed by arrows, called here as “spot d” and “spot c”. The five reconstructed slices equally spaced by 2 μm in the Z direction, with 2 × 2 × 2 μm^3^ of voxel size, are shown for two different energies: (**b**) 7105.9 eV (before the iron K absorption edge), and (**c**) 7144.5 eV (around the Pyrite maximum). (**d**) A XANES spectrum averaged over 8 voxels which are indicated in (**c**), compared to three different measured standards: metallic iron, pyrite^47^ and hematite. (**e**) The local XANES spectra of the 8 voxels indicated in (**c**), which correspond to 5 different hotspots, **a**,**b**,**c**,**d** and **e**.

As a proof of concept, we subsequently applied μED-XAS tomography to investigate the spatial distribution of iron in 1.88 Ga microfossils (about 5 to 10 µm in size) from the Gunflint Iron Formation at Schreiber Beach, Ontario, Canada. These fossils are considered key examples of well-preserved photosynthetic bacteria that may, or may not, have been directly tied to Fe(II) oxidation at the time of deposition. Debate is actively ongoing in terms of whether the presence of Fe(III) in these samples is due to primary metabolic activity of microorganisms comprising the original sediment, or whether the Fe(III) was formed later during burial diagenesis^35^. In this regard, determining the actual speciation of iron in these sedimentary rocks at high spatial resolution is absolutely critical to address this debate. Therefore, the distribution of the iron species within Gunflint samples (as shown in Fig. 5a) was analysed by μED-XAS tomography in a similar manner to the model system containing the different iron mineral standards. Five slices were scanned, spaced 2 µm from each other in the Z direction, as illustrated in Fig. 5a. The used step size in the X direction was 2 µm, and 90 projections were acquired over a θ angular range of 180°. The reconstructed five slices are shown in Fig. 5b for the energy of 7105.9 eV, and in Fig. 5c for the energy of 7144.5 eV. The first energy corresponds to the region of XAS spectra below the Fe K edge, and the second one to the highest observed absorption signal of the sample.

A heterogeneous distribution of iron is observed within the sample. The iron distribution displays Fe-rich spots with rounded shapes and sizes typically ranging from 4–8 µm. The fossil is partially transparent, so that the black spots visible in Fig. 5a are not necessarily close to the surface of the specimen. The Fe K edge XANES can be detected in these spots. The eight spectra, each measured within one single voxel of the five spots richest in iron (shown in Fig. 5c), are, to a first approximation, of similar shape and noise level, as shown in Fig. 5e. The differences between these spectra in the pre-edge region are close to the noise level. Interestingly, each spectrum resembles the pyrite reference spectrum^47^, particularly when comparing the spectrum obtained by averaging these eight spectra (over eight 2 × 2 × 2 µm^3^ voxels) to that of pyrite, as shown in Fig. 5d.

**Figure 6:**
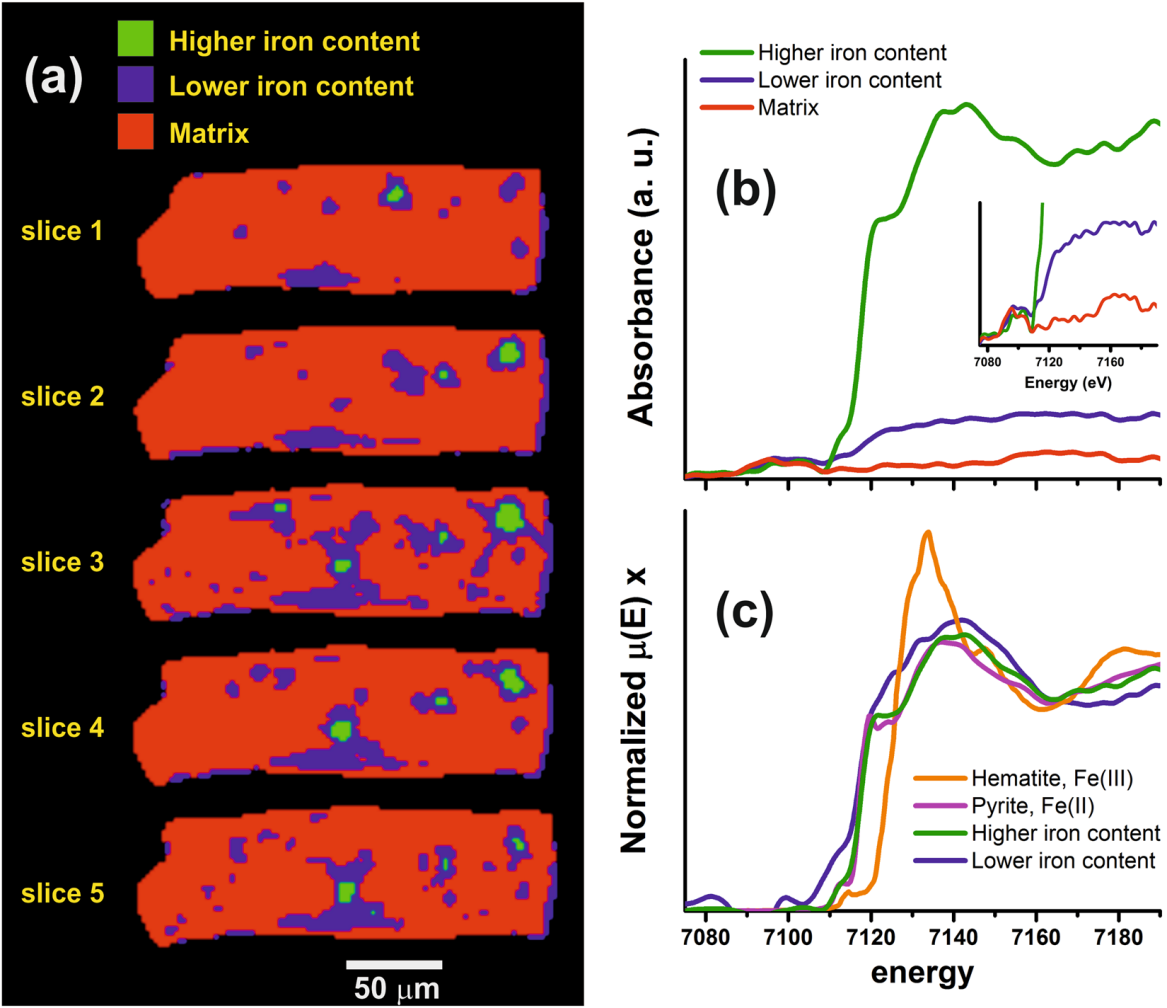
(**a**) Spatial distribution of the higher and lower iron content regions, and the matrix region, and (**b**) the average XANES spectra these three regions. (**c**) XANES spectra of the hematite and pyrite references, and of the average signal of the higher and lower iron content, after subtract the signal from the matrix.

With respect to the Gunflint microfossils, it has been suggested that the filaments enclosed by tubular coverings are reminiscent of modern Fe(II)-oxidizing bacteria^32^, while the spheroid-like vesicles that are a few microns in size are reminiscent of cyanobacteria^31,48^. In the case of the filaments, we mapped three different regions: (i) the matrix where no iron was detected; (ii) low iron domains; and (iii) regions of high iron content corresponding to the spheroid-like hotspots. The low Fe region, domain (ii), is defined with a threshold of 10 percent of the threshold absorption edge defined for the higher iron content, domain (iii). The five reconstructed slices were segmented and the iron content distribution displayed in Fig. 6a. We demonstrate that the hotspots are, in fact, centred in large clumps from which a few elongated Fe-poor zones radiate. A good resemblance with the pyrite spectrum is also observed for the hotspots. No clear signal in XANES spectra is observed by single voxel analysis in the lower iron content region. To investigate the iron speciation of the hotspots, the relative XANES spectra were averaged over the entire probed sample, as shown in Fig. 6b. As highlighted in the inset of Fig. 6b, the features of the matrix XANES signal are observed in both lower and higher iron content XANES spectra before the absorption edge and, also, after the absorption edge for the lower iron content XANES spectrum. The origin of these features is likely to originate from non-ideal beam conditions and/or small-angle X-ray scattering effects which inhibit proper I0 normalization. After subtracting the matrix signal from both iron content XANES spectra, a better agreement develops with the pyrite XANES reference spectrum for the higher iron content (Fig. 6c). By applying the same correction for the lower Fe content region, the spectrum is less similar to the pyrite XANES spectrum than the higher iron content one (Fig. 6c).

**Figure 7:**
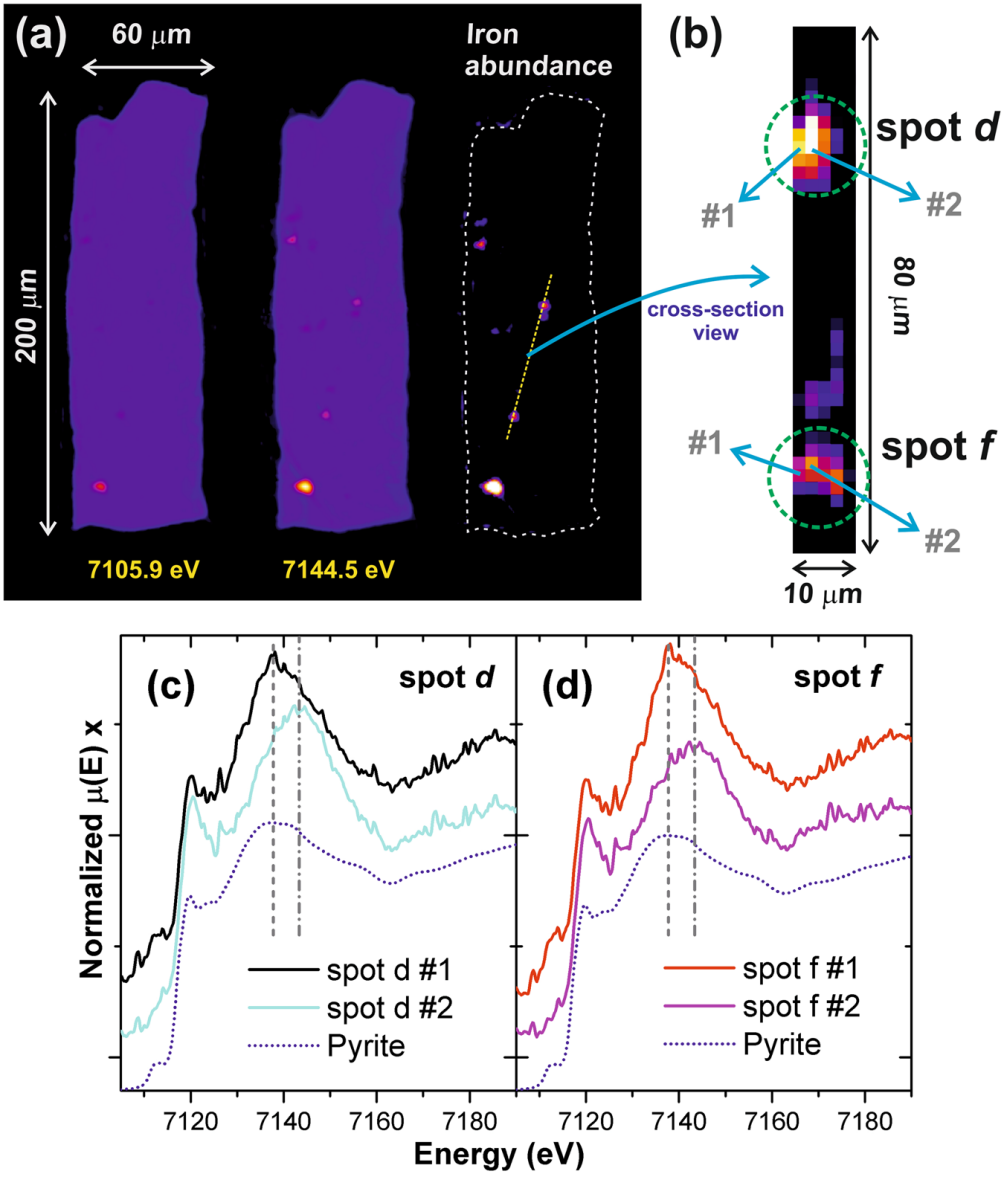
(**a**) The reconstructed slice 5 for the energies of 7105.9 eV and 7144.5 eV, and, the difference between them, highlighting the abundance of iron; along the dashed line indicated in this map, (**d**) a vertical cross-section cut (over the five slices), where the position of spot d and spot f are indicated. The local single voxel XANES spectra from spots (**c**) d and (**d**) f are the same ones shown in Fig. 5e, after matrix signal subtraction.

Although the mismatch between spectra may reflect the limits of the μED-XAS tomographic technique for low concentration specimens, we believe that it more likely points to the presence of a different Fe species. To test the latter, a more detailed analysis of the spectra of spots *d* and *f* (as shown in Fig. 5e) is illustrated in Fig. 7. A vertical cross-section showing the abundance of iron over the dashed line indicated in Fig. 7a is shown in Fig. 7b. In this last image, the positions of spots *d* and *f* are pointed out, as well as the location of the voxels corresponding to the spectra shown in Fig. 5e. The spectra from spots *d* and *f* shown in Fig. 7c,d are the same as those shown in Fig. 5e after subtracting the signal of the matrix, shown in Fig. 6b. In both of these spots, distinct post-edge features are observed; a shift of about + 5–6 eV of the white line is observed for spectra #2. This may indicate differences in the iron coordination along single hotspots. Similar features are observed only on a few point spectra across the scanned region, with the overall average spectrum remaining very similar to the pyrite. In short, we demonstrate that the hot spots associated with the filaments are associated with pyrite, possibly with small variations in stoichiometry and/or the presence of impurities in solid solution, which could explain deviations from the pyrite XANES signature^49,50^. Therefore, despite the Gunflint microfossils being mere tests for the veracity of this technique, what we have shown is that it is indeed possible to obtain useful insights into iron speciation.

The significant high noise level on the obtained XANES Fe K edge spectra, observed in Fig. 5e for example, is due to the relatively low iron concentration detected in transmission mode. An energy dependent high resolution tomography technique, so called XANES Tomography^26^, can be used, but it is subject to the same low concentration limitations. Unsurprisingly, the analysis in transmission mode is always limited to samples with relatively high concentrations. Better data quality could be acquired in fluorescence mode for low concentrations of the element of interest. However, as far as we are aware, 3D full XANES spectra characterization in fluorescence mode with sufficiently high spatial resolution has not yet been reported. An interesting approach is the confocal 3D μXANES method, which in principle resolves XANES spectra measured in fluorescence mode, but with a spatial resolution of about 15–20 µm^51^. This resolution limits the spectrum of materials that could potentially be investigated with this technique due to self-absorption effects, which distort the XANES spectra. Another interesting approach, reported by James and co-authors^22^, is the use of a combination of full 3D XRF tomography with 2D XANES tomography in fluorescence mode to provide the volumetric element distribution and the Cu chemical state spatial distribution for a given slice of the specimen, respectively. A spatial resolution of 2 µm is achieved over an object of about 100–200 µm in size, but since scanning tomography fluorescence XANES demands scans in three dimensions (angle, lateral sample displacement and energy), a full 3D analysis remains very challenging despite important advances in detector technology and in higher count rate efficiency^52^. An interesting alternative would be collecting data in fluorescence mode using the TurboXAS technique^53^, which uses the same scanning tomography setup as proposed in the present work. An example of application of TurboXAS for 2D hyperspectral mapping was shown on metamorphic rocks^54^. Extending this approach to 3D hyperspectral mapping would open new perspectives especially in the investigation of lower pyrite content bacterial microfossil samples (since pyrite is formed during fossilization processes), which might facilitate a better understanding of the metabolic processes of Precambrian microbial life.

## Conclusions

In the present work, we demonstrate the sensitivity and the high spatial resolution (3 µm) of μED-XAS tomography to non-destructively extract 3D speciation information from samples of interest. Specifically, we identify the chemical speciation from different iron mineral standards, and use the technique to identify, for the first time ever, possible differences in the coordination geometry around iron along single 5 to 10 µm microfossils of the 1.88 billion years old Precambrian microfossils from the Gunflint Iron Formation in Canada. Based on these preliminary results, this study will be continued by 2D/3D sub-micron XRF, XRD and XAS analyses to investigate the hypothesis of “metabolic” vs diagenetic iron distributions. The development of this type of analysis can benefit a wide range of applications and research studies, for instance in geology^9,54^, solid oxide cells^44,55,56^, fluid catalytic cracking microparticles for petroleum industry^20,57,58^, degradation processes in nuclear fuel rods^59^, archaeology and cultural heritage^60,61^, planetary science^62^ and paleontology (current results). Given its significantly reduced acquisition time, the present method can also be used as a powerful screening technique to quickly focus on 3D sample speciation heterogeneities which can be further exploited at higher spatial/energy resolutions or lower concentrations by traditional XAS or tomography techniques.

## Methods

### General

We have used iron-mineral standards and associated experimental data obtained at SLS by XAS and full field absorption microtomography to produce a set of 3D speciation imaging. The method of μED-XAS tomography has a resolution of 3 μm, is robust, and could be developed towards higher resolution on the few ED-XAS synchrotron setups worldwide, down to about 1 μm^29^. Absorber concentrations are limited to typically tens of at.%, as usual in transmission XAS – this number varies depending on the nature of the matrix and level of detail that needs to be resolved. The method could also be developed towards higher detection limit by using the TurboXAS technique^53^.

### Experimental Details

The μED-XAS tomography experiments were conducted at the ID24 beamline at the European Synchrotron Radiation Facility (ESRF, Grenoble, France)^30^. A spot size of about 5.5 × 2.5 (H × V) µm^2^ was obtained by horizontally focusing using a bent Si (111) crystal in Bragg geometry and vertically by a mechanically bent mirror^42^. There are several contributions to spot size, including; (1) the intrinsic contribution from dynamical diffraction (≈2 µm FWHM), and (2) the crystal shape and slope error (depends on the crystal manufacturing process). There are also other minor contributions from (1) the penetration depth effect of X-rays in the curved crystal (at 7 keV it is negligible), and (2) from the demagnification of the source, which is negligible at this beamline. On ID24, the resolution limit in the horizontal direction is of the order of 2–3 µm FWHM. In the vertical direction, the optical scheme of the beamline provides a vertical spot of the order of 2–3 µm^29,30^. A polychromatic energy range of about 400 eV is obtained in this experiment, although in optimal conditions a range of 900 eV can be achieved at the Fe K-edge using a Si(111) crystal. The intensity transmitted by the sample is detected by a fast, low-noise, high-dynamic-range X-ray position-sensitive detector^43^. For the tomographic scans, the used acquisition time was 100 ms per spectrum. In this work, 1300 pixels out of the 2048 were used to analyse an energy range of about 400 eV around the Fe K-edge (7.112 keV). Each sample was positioned in the X-ray beam focal spot, mounted on a motorized rotational stage. The tomography data acquisitions were performed by collecting XAS spectra in two dimensions: X and θ, as illustrated in Fig. 1. XAS spectra were collected at several tens of lateral sample X positions, all along the samples’ lateral sizes, equally spaced by 1 to 2 µm, and at 90–180 θ positions equally spaced as well, over an angle range of 180°. The sample is analysed slice by slice, where each slice measurement consists in a 2D X by θ scan. Each slice is measured in about one to two hours. Sufficient adjacent slices have to be measured in order to obtain the full 3D sample analysis.

The model system with iron containing compounds was prepared as a mixture of of (i) staurolite (an iron aluminium silicate containing Fe^2+^), (ii) magnetite (an iron oxide with Fe^2+^ and Fe^3+^), (iii) hematite (another iron oxide mix of Fe^2+^ and Fe^3+^) and (iv) hypersthene (an iron aluminium silicate containing Fe^2+^). All the iron compounds were gently hand-ground using a pestle and mortar in order to obtain grains of about 2–30 μm in size. The powders were mixed together with an organic UV-hardening glue (Norland Optical Adhesive 68). In the next step, a 300 µm diameter silica glass capillary with 10–20 µm wall-thickness was filled with the mixture, to be later hardened by UV irradiation. An optical microscopy image of the sample is shown in Fig. 2.

All the standard reference compounds were individually pre-characterized through X-ray absorption spectroscopic measurements at the microXAS beamline of the Swiss Light Source (SLS, PSI, Villigen, Switzerland). The XAS measurements were performed simultaneously in absorption and fluorescence modes, at room temperature, using a monochromatic beam. The energy of the X-ray beam was selected using a fixed-exit monochromator equipped with a pair of Si(111) crystals. The transmitted signal was collected using a standard silicon diode. The fluorescence signal was detected with a single-element Si detector (Ketek).

The model system containing iron compounds was post-characterized using full field absorption X-ray synchrotron microtomography on the SLS TOMCAT beamline^63^. The beamline was operated at an energy of 20 keV obtained from the Multilayer (Ru/C stripe, 10^−2^ bandwidth), and the low energies were cut by a 250 µm aluminium filter. The sample was placed on the high resolution microtomographic end-station at 25 m from the source. Data were collected using an indirect image detector consisting of a 20 µm thick LuAg:Ce scintillator, a 20X objective and a CCD-based camera (PCO Edge 5.5, 2560 × 2160 pixels). The detector was thus operated with an effective pixel size of 0.325 µm. The exposure time was set to 0.2 seconds and 2001 projections of the fixed specimen were taken during rotation over a range of 180°, as well as 10 dark field and 40 flat field images. The sample-detector distance was as small as possible to avoid phase contrast. The image reconstructions were obtained using the GridRec algorithm with a Parzen filter^64^, and then the images were converted to 8 bits TIF images.

## Electronic supplementary material

Supplementary information
