# Supplementary material for: 2D/3D Microanalysis by Energy Dispersive X-ray Absorption Spectroscopy Tomography
Source: Sci Rep. 2017 Nov 28;7:16453. doi: 10.1038/s41598-017-16345-x (PMC5705590; doi:10.1038/s41598-017-16345-x)
Supplement: Supplementary file 1 — Supplementary information [file 41598_2017_16345_MOESM1_ESM.pdf]

SUPPLEMENTARY INFORMATION

2D/3D Microanalysis by Energy Dispersive X-ray Absorption Spectroscopy Tomography

Dario Ferreira Sanchez[a,*], Alexandre S. Simionovici[b], , Laurence Lemelle[c], Vera Cuartero[d], Olivier Mathon[d], Sakura Pascarelli[d], Anne Bonnin[a], Russell Shapiro[e], Kurt Konhauser[f], Daniel Grolimund[a], Pierre Bleuet[g]

[a]*Paul Scherrer Institut, CH-5232 Villigen PSI, Switzerland*
[b]*ISTerre, UGA, CNRS, Observatoire des Sciences de l'Univers, CS 40700, 38058 Grenoble, France*
[c]*LGL-TPE, Univ. de Lyon, CNRS, Ecole Normale Supérieure de Lyon, Lyon, France*
[d]*ESRF-The European Synchrotron, 71, Avenue des Martyrs, Grenoble, France*
[e]*Geological and Environmental Sciences Dept., CSU Chico, Chico, CA, USA*
[f]*Dept. of Earth and Atmospheric Sciences, Univ. of Alberta, Edmonton, AB, Canada*
[g] *Univ. Grenoble Alpes, F-38000 Grenoble, France ; CEA/Liten, MINATEC Campus, 17 rue des Martyrs, 38054 Grenoble Cedex 9, France*
* *Dario.Ferreira@psi.ch*

Resolution

The resolution of 3 μm was measured by analysing the absorbance intensity along sharp edges of the analysed samples, as illustrated in Supplementary Figure 1a-b and, also, through Fourier Ring Correlation[46], by analyzing several reconstructed images at different contiguous energies of the same sample slice, resulting in a resolution of 3.8 μm, 3.8 μm and 3.2 μm for three different thresholds methods as follows: fixed 1/7, Half-bit and three sigmas, respectively.

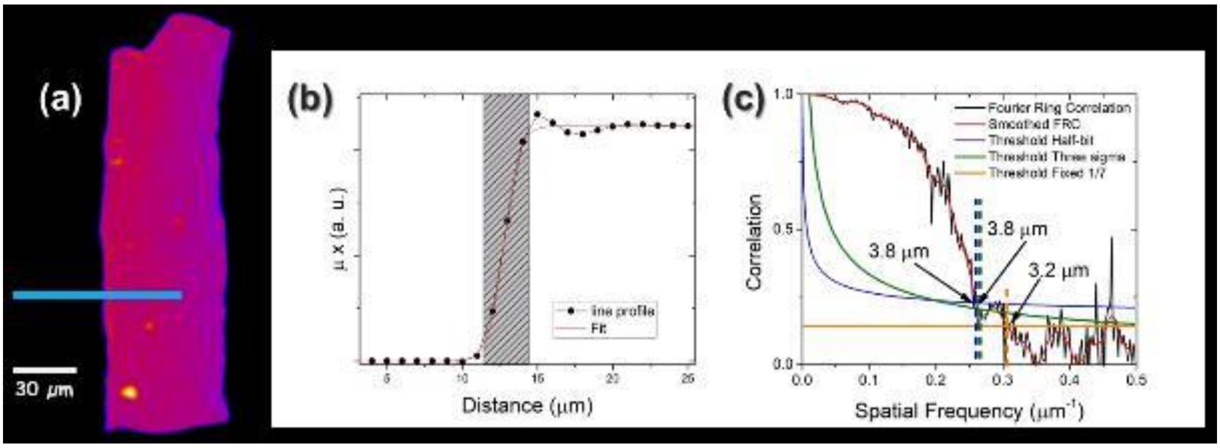

Supplementary Figure 1: (a) Reconstructed slice of the microfossil sample. (b) The intensity profile along the blue line shown in (a). Fourier Ring Correlation with images corresponding to different energies, which can essentially be considered as independent measurements; three different thresholds methods are considered: fixed 1/7, Half-bit and three sigma[46], which as resolution values 3.8 μm, 3.8 μm and 3.2 μm, respectively.

The various energies do not necessarily intersect the same region within the specimen due to the non-ideal focusing conditions (non-achromatic focusing), as illustrated in Supplementary Figure 2, which is mainly due to the small divergences from a perfect elliptical shape of the polychromator as

well as small fluctuations from the zero slope errors. These values may differ by up to 10 µm. This, with the fact that the various energies transit the specimen at different angles, is the reason why it is necessary to apply a rigid-body motion (rotation and translation), to obtain a matching of the all reconstructed images (for the different energies) with respect to the first one by using image correlation methods for a given slice (a given Z sample position).

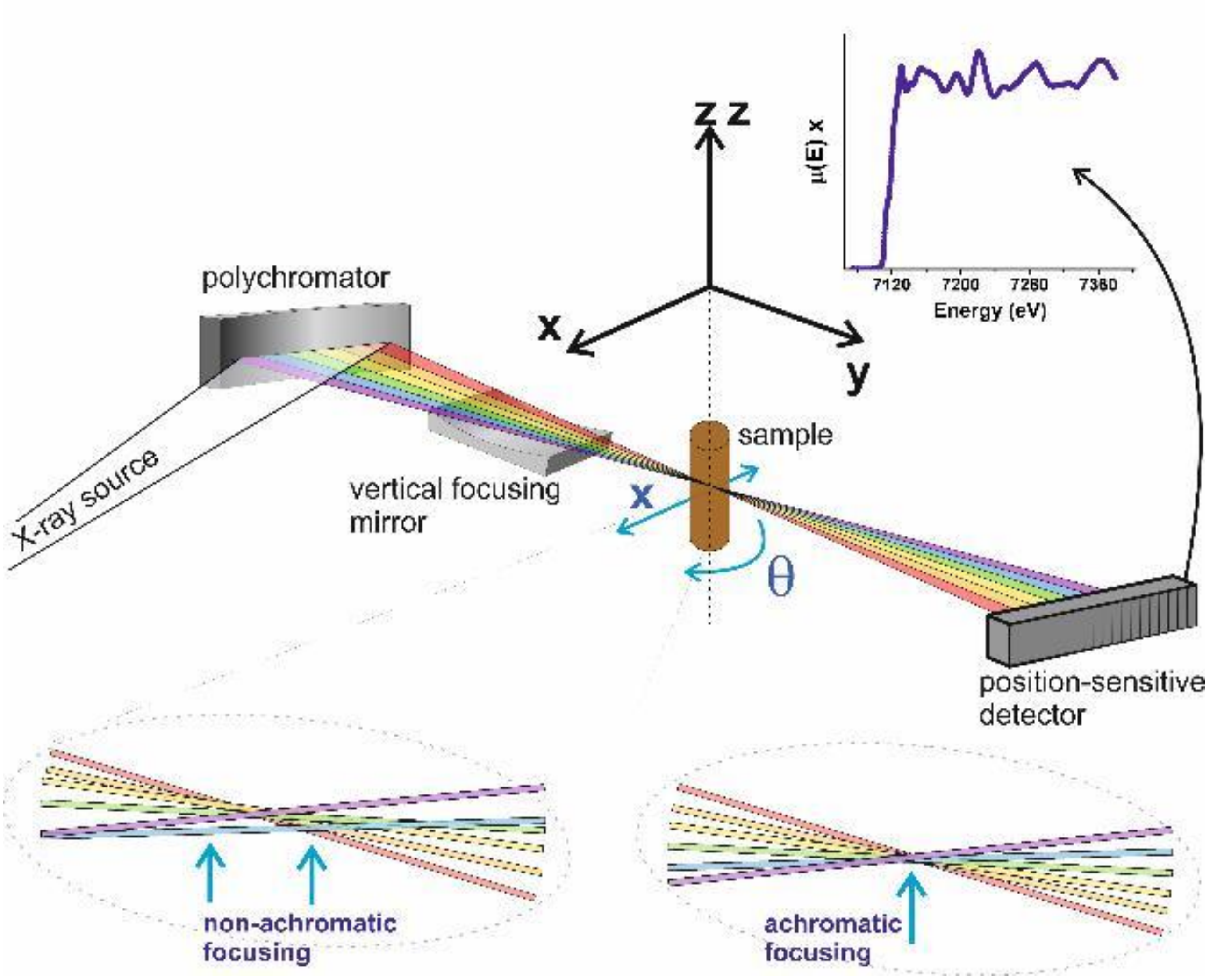

Supplementary Figure 2: Illustration of the non-ideal focusing conditions effect, which leads to a non-achromatic focusing.

Chemical Contrast

In Supplementary Figure 3 is shown the same analysed slice in **Error! Reference source not found.**. In addition to the comparison between the reconstructed images for the energies 7106.9 eV and 7125.4 eV, also the comparison between images corresponding to energies 7131.0 eV and 7144.9 eV are shown. A first qualitative inspection in the images Supplementary Figure 3c-f indicate the different chemical states of these two particles. Comparing single voxel XANES

spectra corresponding to particles 1 and 2, the compounds are readily identified as hypersthene and magnetite, respectively.

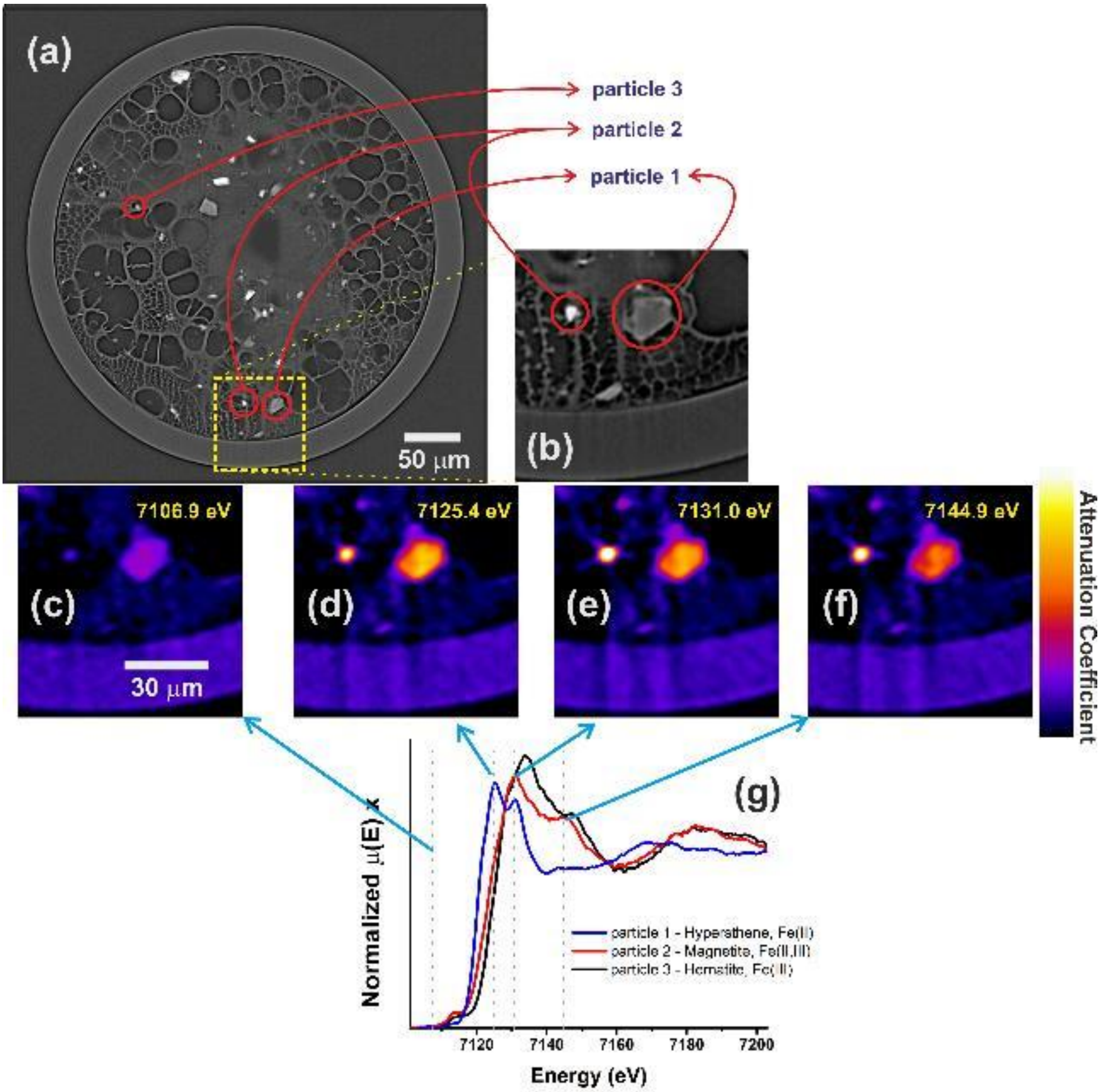

Supplementary Figure 3: For the glass capillary sample containing several iron species, (a) a reconstructed slice from the full field microtomography experiments using an X-ray energy of 20 keV. (b) A detailed view corresponding to the dashed rectangle in (a), and, for this same region, reconstructed images through µED-XAS tomography different energies: (c) 7106.9 eV, (d) 7125.4 eV, (e) 7131.0 eV and (f) 7144.9 eV. In (g), single voxel XANES spectra corresponding to particles 1 to 3, indicated in (a).
